# Aflatoxin Exposure-Caused Male Reproductive Toxicity: Molecular Mechanisms, Detoxification, and Future Directions

**DOI:** 10.3390/biom14111460

**Published:** 2024-11-17

**Authors:** Dongyun Ye, Zhihui Hao, Shusheng Tang, Tony Velkov, Chongshan Dai

**Affiliations:** 1 Department of Obstetrics and Gynecology, Ezhou Central Hospital, Hubei University of Science and Technology, Ezhou 436000, China; 2 National Key Laboratory of Veterinary Public Health and Safety, College of Veterinary Medicine, China Agricultural University, Beijing 100193, China; 3 Key Biology Laboratory of Chinese Veterinary Medicine, Ministry of Agriculture and Rural Affairs, Beijing 100193, China; 4 Department of Pharmacology, Biodiscovery Institute, Monash University, Clayton, VIC 3800, Australia

**Keywords:** AFB_1_, mycotoxins, reproductive toxicity, molecular mechanisms, detoxification

## Abstract

Widespread endocrine disorders and infertility caused by environmental and food pollutants have drawn considerable global attention. Aflatoxins (AFTs), a prominent class of mycotoxins, are recognized as one of the key contributors to environmental and food contamination. Aflatoxin B_1_ (AFB_1_) is the most potent and toxic pollutant among them and is known to cause multiple toxic effects, including neuro-, nephro-, hepato-, immune-, and genotoxicity. Recently, concerns have been raised regarding AFB_1_-induced infertility in both animals and humans. Exposure to AFB_1_ can disrupt the structure and functionality of reproductive organs, leading to gametogenesis impairment in males, subsequently reducing fertility. The potential molecular mechanisms have been demonstrated to involve oxidative stress, cell cycle arrest, apoptosis, inflammatory responses, and autophagy. Furthermore, several signaling pathways, including nuclear factor erythroid 2-related factor 2; NOD-, LRR-, and pyrin domain-containing protein 3; nuclear factor kappa-B; p53; p21; phosphoinositide 3-kinase/protein kinase B; the mammalian target of rapamycin; adenosine 5′-monophosphate-activated protein kinase; and mitochondrial apoptotic pathways, are implicated in these processes. Various interventions, including the use of small molecules, Chinese herbal extracts, probiotic supplementation, and camel milk, have shown efficacy in ameliorating AFB_1_-induced male reproductive toxicity, by targeting these signaling pathways. This review provides a comprehensive summary of the harmful impacts of AFB_1_ exposure on male reproductive organs in mammals, highlighting the potential molecular mechanisms and protective agents.

## 1. Introduction

Infertility has been regarded as a significant public health concern by the World Health Organization (WHO) and it affects around 48 million couples and 186 million individuals globally [1]. Experimental and epidemiological studies have indicated associations between diminished male fertility and exposure to various environmental toxins, such as heavy metals, cigarette smoke, microplastics, plasticizers, persistent organic pollutants, and mycotoxins [2,3]. In addition, infections caused by viruses or bacteria are also one of the most important emerging causes of infertility [4,5]. Mycotoxins are secondary metabolites synthesized by filamentous fungi, including genera such as *Fusarium*, *Aspergillus*, and *Penicillium* [6]. Currently, around 500 mycotoxins have been reported in the natural environment, including fumonisins (FBs), T-2 toxin, deoxynivalenol (DOX), HT-2 toxin, aflatoxins (AFTs), ochratoxin A (OTA), zearalenone (ZEN), cytochalasins, penicillic acid, citrinin, fusarin C, patulin, and tenuazonic acid [7,8]. The Food and Agriculture Organization estimated that approximately 25% of global food crops were contaminated by mycotoxins before 1985 and this figure has risen to approximately 60–80% in 2020, based on improvements to analytical methods and the impact of climate change [8]. Importantly, these mycotoxins can induce a range of toxic effects and contribute to severe health conditions in both humans and animals [6,9]. Overall, mycotoxin contamination of food and the environment pose significant public health and safety challenges worldwide.

AFTs represent one of the most significant classes of mycotoxins, characterized by their high toxicity. Chemically, AFTs are difurocoumarolactones (difurocoumarin derivatives) [10]. Presently, AFTs remain a substantial food safety concern, particularly in developing nations [11]. It has been reported that AFTs comprise 21 known members, including aflatoxin B1 (AFB_1_), AFB_2_, aflatoxin G1 (AFG_1_), AFG2, and aflatoxicol M_1_ (AFM_1_) [7]. Among these, AFB_1_ is recognized as the most toxic and it has been shown to induce geno-, gastrointestinal, immuno-, nephro-, hepato-, neuro-, cardio-, and carcinotoxicity in domestic animals and rodents [11,12,13,14,15,16,17,18,19,20]. Epidemiological investigations indicate that exposure to AFTs is associated with a range of chronic diseases, including cancer, neurodegenerative disorders, chronic liver disease, cardiovascular disease, and immunological dysregulation [21,22,23,24]. Recent studies suggest that exposure to AFB_1_ may result in considerable reproductive toxicity in male animals [25,26]. For instance, in a rat model, AFB_1_ treatment at a dose of 50 μg/kg body weight per day, via oral exposure for 56 consecutive days, significantly decreased testicular function and both the quality and quantity of sperm [27]. Owumi et al. reported that oral exposure to AFB_1_ at the same dose for 28 consecutive days resulted in marked testicular tissue injury, characterized by a severe imbalance in the redox system and inflammatory responses in male rats [25]. Several studies, to date, have explored the potential molecular mechanisms underlying AFT exposure-induced reproductive toxicity in male animals, which involve oxidative stress, cell cycle arrest, apoptosis, autophagy, and inflammatory responses [25,26,27]. This review encompasses the most significant articles concerning AFT exposure-induced male reproductive toxicity from the PubMed, Web of Science, and Scopus databases, published between 1 January 1980 and 1 October 2024. The keyword combinations employed for the literature search included: aflatoxins (or AFTs) and male reproductive toxicity (or male infertility), or aflatoxin B_1_ (or AFB_1_) and male reproductive toxicity (or male infertility). The harmful effects of AFT exposure on the reproductive organs of male mammals, the molecular mechanisms, and potential protective agents, were summarized. We hope this review offers valuable insights and stimulates further studies and broader discussions aimed at developing effective protective agents against AFT exposure-induced reproductive toxicity in male mammals.

## 2. Effects of AFB_1_ Exposure on Hypothalamic–Pituitary–Gonadal Axis and Hormone Production

In mammals, the hypothalamic–pituitary–gonadal (HPG) axis mediates the production of hormones, which mainly govern the reproductive function and fertility [28]. This reproductive axis is controlled at multiple levels, such as in the brain and pituitary gland. This allows for either the promotion or the inhibition of gonadal sex steroid secretion and function. Any disruption in any part of the HPG axis may lead to reproductive problems [28]. The gonadotropin-releasing hormone (GnRH; also named the luteinizing hormone-releasing hormone [LHRH]) is secreted by the hypothalamus and has an impact on the secretion of pituitary gonadotropins, like the luteinizing hormone (LH) and follicle-stimulating hormone (FSH). Thus, it plays a crucial role in the physiological aspects of mammalian reproduction [29]. Earlier research showed that when male chickens were exposed to AFTs (which were made up of 85% AFB_1_, 13% AFB_2_, and 2% AFG_1_) at a concentration of 10 ppm (equivalent to 10 μg/mL), their plasma LH concentrations increased significantly. Also, researchers discovered that AFT exposure did not influence the anterior pituitary gland’s LH secretory capacity, but changed the testicular response to exogenous GnRH [30]. This research demonstrates that due to chronic dietary exposure, AFTs can cause distinct endocrine changes in male chickens [30]. Rotimi et al. reported that early life exposure to AFB_1_ at concentrations of 0.5 and 5 mg/kg of feed, starting two weeks before mating and continuing throughout pregnancy until three weeks after delivery, had a marked impact on the offspring’s hormonal levels [31]. In all groups treated with AFB_1_, the plasma LH levels and testosterone levels were significantly decreased [31]. Testosterone biosynthesis occurs in the testes’ Leydig cells in response to LH and a decrease in testosterone is positively related to lower LH levels [32]. Recently, Owumi et al. showed that oral exposure to AFB_1_ at 50 μg/kg of body weight for 28 days seriously damaged hypothalamic tissues. This led to a decrease in serum FSH, LH, and testosterone levels, while prolactin levels increased, in male rats [33]. In order to produce testosterone, cholesterol is first transported into the cytoplasm of Leydig cells by scavenger receptor class B member 1 (SCARB1), and then further transferred into the mitochondrial inner membrane by the steroidogenic acute regulatory protein (STAR), where it is catalyzed to produce pregnenolone by the cytochrome P450 family 11 subfamily A member 1 (CYP11A1), and further testosterone is produced by a series of androgen synthases, such as CYP17A1, HSD3B1, and HSD17B3, in the smooth endoplasmic reticulum of Leydig cells [26,34]. Chen et al. found that AFB_1_ treatment could significantly decrease the expression of SCARB1, CYP11A1, CYP17A1, 3β-hydroxysteroid dehydrogenase (HSD3B1), and 17β-hydroxysteroid dehydrogenase 3 (HSD17B3) mRNAs and proteins in primary rat adult Leydig cells and rat testis tissues [34]. Similar results were also found by Ijaz and colleagues [27]. These findings indicate that AFB_1_ disturbs the testosterone biosynthesis function of Leydig cells through downregulating the expression of certain crucial androgen synthesis-related proteins. Additionally, Zhang et al. found that daily oral treatment with AFB_1_ at 200 μg/kg of body weight for 28 days significantly reduced the expression of hormone synthesis-related proteins, including STAR and CYP11A1 [26]. Moreover, researchers showed in docking simulations that AFB_1_ could directly interact with the STAR protein and it had a high binding affinity compared to the natural ligand cholesterol [32]. This interaction might interfere with the transport of cholesterol into mitochondria and, thus, reduce steroidogenesis. This finding clarifies the molecular mechanisms of STAR inhibition, according to which AFB_1_ exposure causes a reduction in testosterone levels.

**Figure 1:**
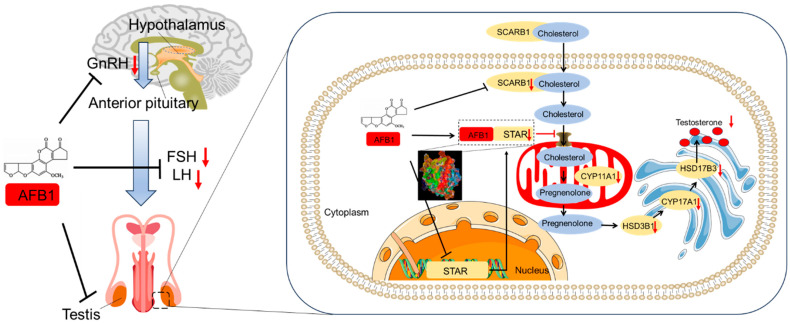
A schematic summary of the deleterious effects of AFB_1_ exposure on the hypothalamic–pituitary–testis axis and the inhibition of hormone production in Leydig cells. Notes: AFB_1_, aflatoxin B_1_; CYP11A1, cytochrome P450 family 11 subfamily A member 1; GnRH, gonadotropin-releasing hormone; LH, luteinizing hormone; FSH, follicle-stimulating hormone; SCARB1, scavenger receptor class B member 1; STAR, steroidogenic acute regulatory protein; HSD3B1, 3β-hydroxysteroid dehydrogenase; HSD17B3, 17β-hydroxysteroid dehydrogenase 3; CYP17A1, cytochrome P450 family 17 subfamily A member 1. 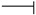 indicates inhibition; 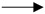 indicates promotion.

In short, the evidence indicates that AFB_1_ exposure can disrupt the HPG axis and inhibit the production of FSH and LH, then reduce the synthesis of testosterone, finally causing reproductive dysfunction and infertility in males. Moreover, AFB_1_ exposure-mediated inhibition of testosterone synthesis in Leydig cells may involve the inhibition of the SCARB1/STAR/CYP11A1 pathway. This may largely be due to the fact that AFB_1_ exposure could inhibit the transcriptional expression of STAR or directly interact with STAR, thus inhibiting the transportation of cholesterol. Figure 1 summarizes the working model of AFB_1_ exposure on the destruction of the hypothalamic–pituitary–testis axis and the production of hormones in Leydig cells. To date, further research into the exact molecular mechanisms of AFB_1_ exposure on each part of the HPG axis and the production of testosterone is urgently lacking.

## 3. Effect of AFB_1_ Exposure on Male Fertility, Reproductive Organs, and Sperm Quality

The prevalence of infertility among couples globally is on the rise. Male-specific fertility problems account for around 40–50% of all infertility cases and up to 2% of men have less-than-optimal sperm parameters [35]. There is a growing amount of evidence suggesting that exposure to mycotoxins is an important risk factor for male fertility [2,35,36]. Epidemiological research has found that the levels of AFTs in infertile men is roughly five times greater than in normal individuals; the average aflatoxin concentration is significantly higher in infertile men (average value: 1.660 ± 1.041 μg/mL) compared to fertile men (average value: 1.041 ± 0.01 μg/mL) [37]. Animal studies have indicated that exposure to AFB_1_ can undermine the integrity of the blood–testis barrier, enabling the toxin to penetrate testicular tissue and leading to abnormal histopathological changes in both testicular and epididymal tissues. This eventually results in a decline in sperm quantity and quality, as well as reproductive dysfunction and infertility [38,39]. Moreover, it was shown that the dysfunction in the blood–testis barrier mediated by AFB_1_ is related to the downregulation of several crucial junction proteins, such as occludin, connexin 43, and N-cadherin [38,39,40]. However, more research is needed to clarify the specific mechanisms through which AFB_1_ disrupts the blood–testis barrier.

The testis is a complex and multi-functional reproductive organ composed of two different areas: seminiferous tubules and interstitial tissue, where Leydig cells are located [41,42]. Enclosed by basement membranes and peritubular myoid cells, the seminiferous tubules contain spermatogonia stem cells, Sertoli cells, and spermatogonia at different stages [42]. Spermatozoa development from spermatogonia requires coordinated interactions between somatic cells and germ cells. Any interference in these intermediate processes or intercellular regulations can affect the reproductive functions of the testes.

Presently, male fertility is on the decline globally, mainly due to sub-par sperm quality [43]. Earlier research has shown that being exposed to AFB_1_ and its derivatives can harm germ cells, Sertoli cells, Leydig cells, and considerably lower gonadotropin levels, such as LH and FSH, as well as testosterone in male animals, including mice, rats, pigs, and sheep [44,45,46]. These detrimental effects ultimately lead to diminished sperm quality and quantity, resulting in compromised reproductive performance and potential infertility. For instance, Ashraf et al. observed that administering AFB_1_ at 100, 200, and 400 ppm in feed over 10 weeks resulted in a dose-dependent decrease in the weight and volume of the testes, causing partial to complete arrest of spermatogenesis in juvenile white leghorn male birds [47]. Furthermore, Supriya et al. reported that exposing male rats to AFB_1_ at 20 and 50 μg/kg of body weight through intramuscular injections for 60 days significantly reduced the weight and indices of the testis and accessory sex organs (cauda epididymis, caput epididymis, prostate) [32]. Lin et al. discovered that acute oral exposure to high doses of AFB_1_ (1 mg/kg of body weight) induced significant morphological changes in the testis, including intracellular vacuolation, reduced vas deferens diameter, and thinning of the spermatogenic epithelium, in male sheep [39]. Additionally, their study showed that AFB_1_ treatment at 50 μg/kg of body weight, through oral administration for 28 days, notably decreased the relative weight of the testes and epididymis, impaired sperm function (i.e., motility, viability, and count), and increased sperm abnormality rates. It is noteworthy that apoptotic and inflammatory responses were evident in the AFB_1_-exposed testicular tissue [25,33]. These findings underscore the disruptive impact of AFB_1_ exposure on the function and development of male reproductive organs, leading to reduced sperm quality, impaired spermatogenesis, and ultimately decreased reproductive performance, including infertility. Despite these observations, the intricate molecular mechanisms involved require further, detailed investigation. Table 1 summarizes the harmful toxic effects of AFB_1_ exposure on the reproductive organs and performance of male animals [25,26,33,38,39,47,48,49,50,51,52,53,54].

## 4. Molecular Mechanisms of AFB_1_-Related Reproductive Toxicity in Males

The potential mechanisms of AFB_1_ exposure-induced injury to male reproductive organs have been investigated across multiple animal studies [25,32,33]. Studies of mouse or rat models have indicated that exposure to AFB_1_ can result in damage to testicular and epididymal tissues via mechanisms like oxidative stress, inflammatory responses, autophagy, and cell apoptosis [25,26,55]. Lately, transcriptomic analysis carried out on the testis tissue of mice demonstrated that AFB_1_ exposure directly gives rise to abnormal alterations in multiple signaling pathways, including p53, p21, nuclear factor erythroid 2-related factor 2 (Nrf2), phosphatidylinositide 3-kinase (PI3K)/protein kinase B (AKT), nuclear factor-kappa B (NF-κB), mitogen-activated protein kinase (MAPK), NLR family pyrin domain containing 3 (NLRP3), AMP-activated protein kinase (AMPK), and the mammalian target of rapamycin (mTOR) pathways [7,19,25,26,27,31,32,33,38,39,40,46,49,50,54,55,56,57,58,59,60,61,62,63]. AFB_1_ can also lead to a decline in fertility; notably, exposure to mice results in testicular bleeding, edema, necrosis, and atrophy. Moreover, decreases in sperm count, viability, quality, and motility were observed [25,27,32,33,44,45,46]. The following sections will discuss in detail the potential molecular mechanisms of AFB_1_ exposure on various cell types in testicular tissues.

### 4.1. AFB_1_-Related Induction of Oxidative Stress

Oxidative stress, which occurs due to an imbalance between pro-oxidant molecules and antioxidant systems, is crucial in the development of subfertility, in both animals and humans. There has been extensive documentation regarding the negative impacts of oxidative stress on sperm quality and function [64,65]. Oxidative stress involves reactive oxygen species (ROS) as the primary pro-oxidant molecules. These ROS include hydrogen peroxide (H_2_O_2_), superoxide anion (O^2•−^), singlet oxygen (^1^O_2_), and hydroxyl radicals (•OH) [66]. Under normal physiological conditions, the intracellular antioxidant system, consisting of vitamin C (VitC), vitamin E (VitE), reduced glutathione (GSH), and diverse antioxidant enzymes, swiftly counteracts ROS and alleviates oxidative stress [65]. But when facing environmental stress, the overproduction of ROS within cells disturbs this equilibrium, causing oxidative harm to various cellular macromolecules, such as lipids, proteins, and DNA [51]. This, in the end, leads to different forms of regulated cell death [67,68,69]. Many studies have indicated that exposure to AFB_1_ results in excessive ROS generation, which brings about oxidative stress damage in the male reproductive system, particularly in testicular and epididymal tissues [25,26,39,44,46,47,70]. Cao et al. discovered that orally administering AFB_1_ at a dose of 0.75 mg/kg of body weight, daily, for 45 days, notably increased the ROS and malondialdehyde (MDA) levels, while significantly reducing glutathione peroxidase (GPX) activity, in mouse testicular tissues [54]. Crucial intracellular antioxidant enzymes, like catalase (CAT), GPX, and superoxide dismutase (SOD), are vital parts of the antioxidant defense system [66]. Normally, intracellular SOD changes O^2•−^ into H_2_O_2_, and then CAT or GPX further catalyzes H_2_O_2_ into harmless substances, namely water (H_2_O) and oxygen (O_2_) [66]. In a rabbit model, researchers noticed that exposing rabbits to AFB_1_ at a low dose (AFB_1_ was mixed in feed with 0.5 mg/kg, meaning about 0.3 μg/kg body weight was consumed daily), for 21 days, significantly increased MDA levels, while considerably decreasing the activities of SOD, CAT, and the total antioxidant capacity (T-AOC) in testicular tissue [40]. Lin et al. reported that acute exposure to AFB_1_ at 1 mg/kg of body weight, through intragastric administration for 24 h, substantially decreased the mRNA expression of GPX3 and SOD2 genes in sheep testicular tissues [39]. Since GPX3 and SOD2 genes code for the enzymatic proteins GPX and SOD, respectively, it shows that AFB_1_-induced oxidative damage in testicular tissues includes the suppression of antioxidant enzyme-related gene expression and activity.

The Nrf2/Kelch-like ECH-associated protein 1 (Keap1) signaling pathway plays a key role reproductive function, by modulating the redox balance and the inflammation of testis tissues [71,72]. When it comes to oxidative damage, Nrf2 is an important transcription factor that controls the expression of numerous downstream antioxidant genes, such as NAD(P)H quinone oxidoreductase-1 (NQO-1), heme oxygenase 1 (HO-1), GPX, CAT, SOD, and glutathione-*S*-transferase (GST) genes [72,73]. In normal conditions, the Nrf2 protein is kept at relatively low concentrations because Nrf2 is continuously ubiquitinated by Keap1, an adaptor part of the Cul3 (Cullin 3)-based ubiquitin E3 ligase complex, and targeted for proteasomal degradation [74]. In stressed conditions, nonetheless, the ubiquitination of Nrf2 by Keap1 is restrained, which enables Nrf2 to get into the nucleus and trigger the expression of its downstream genes [74]. It has been demonstrated that Nrf2 knockout rats are highly sensitive to AFB [75]. Following AFB_1_ exposure, there is increased expression of Keap1 at the mRNA level in the testicles of rats [27]. These data also suggested that the transcriptional inhibition of Nrf2 by AFB_1_ exposure is partly attributed to the regulation of Keap1. Huang et al. found that AFB_1_ exposure at 0.75 mg/kg of body weight per day, for 30 days, significantly decreased nuclear Nrf2 protein expression and the mRNA levels of the downstream targets HO-1, NQO-1, CAT, and SOD1, in mouse testes [61]. Ijaz et al. reported that AFB_1_ exposure at 50 μg/kg of body weight per day, via oral administration for 8 weeks, significantly downregulated the mRNA expressions of Nrf2 and its downstream genes, including CAT, SOD, GPX, HO-1, glutathione reductase (GSR), and GST [27]. It is well-known that GST and GSH are two critical endogenous detoxifying agents of AFB_1_. GSH could, through the formation of AFB_1_–GSH conjugation, serve as the main route for the detoxification of AFB_1_. The GST isoenzyme brings about detoxification via catalyzing exo-AFBO to form a combination with GSH [7,76]. The upregulation of GSH and GST through the activation of Nrf2 contributed partly to its protective effects. Moreover, numerous studies have indicated that the Nrf2/HO-1 pathway, which is a crucial endogenous antioxidant mechanism, has a protective function against AFB_1_-induced hepatotoxicity, nephrotoxicity, and neurotoxicity [77,78]. All the time, lycopene, which is an inductor of Nrf2, can activate Nrf2 and efficiently relieve oxidative stress, mitochondrial dysfunction, and cellular apoptosis, in the testicular tissues of mice brought about by AFB_1_ exposure [61].

**Figure 2:**
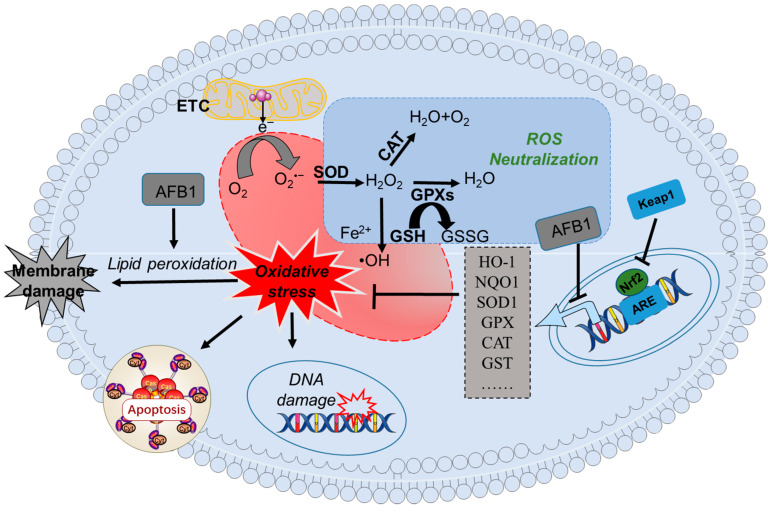
A schematic summary of AFB_1_-induced oxidative stress processes in testicular tissues. Notes: AFB_1_, aflatoxin B_1_; GSH, reduced glutathione; GPX, glutathione peroxidase; GST, glutathione S-transferase; H_2_O_2_, hydrogen peroxide; HO-1, heme oxygenase-1; Keap1, Kelch-like ECH-associated protein 1; Nrf2, nuclear factor erythroid 2-related factor 2; NQO1, NAD(P)H quinone oxidoreductase 1; O^2•−^, superoxide anion; •OH, hydroxyl radicals; ROS, reactive oxygen species; CAT, catalase; ETC, mitochondrial respiratory chain complex; SOD, superoxide dismutase.

In summary, oxidative stress damage is highly important in terms of the male reproductive toxicity caused by AFB_1_ exposure. As illustrated in Figure 2, existing evidence shows that the oxidative stress induced by AFB_1_ is mainly due to the induction of lipid peroxidation, the disruption of the body’s antioxidant system, and the inhibition of the Nrf2 pathway.

### 4.2. AFB_1_-Related Mitochondrial Dysfunction and Mitochondrial Apoptotic Pathways

The mitochondria act as “power generators” within the cell and represent the main origin for the generation of adenosine triphosphate (ATP) [79]. Apart from generating energy, mitochondria also carry out numerous other vital functions. These include the control of the redox state, signaling routes, and different kinds of cell death. Moreover, mitochondria can be both generators and receptors of ROS [79]. The overproduction of ROS can disturb the normal functioning of mitochondria, causing harmful impacts that reduce the cell’s survival ability [79]. In germ cells, mitochondria are the most crucial organelle for energy production. Significantly, the connection between mitochondrial function and sperm parameters has been emphasized in multiple studies [80,81]. Huang et al. employed a mouse model to show that AFB_1_ exposure at a dosage of 0.75 mg/kg of body weight daily, for 30 days, seriously damaged the mitochondrial ultrastructure and decreased the quantity of mitochondria in spermatogonia, Leydig cells, and Sertoli cells, within testicular tissue treated with AFB_1_ [61]. Another piece of research indicated that when exposed to AFB_1_ at doses of 0.375, 0.75, and 1.5 mg/kg of body weight, each day for 30 days, damage to the mitochondrial ultrastructure in Leydig cells and germ cells could be triggered, in a dose-related fashion. Noticeable irregularities in the mitochondrial cristae and matrix were seen in all the groups treated with AFB_1_ [59]. Consistently, an in vitro model created by Komsky-Elbaz et al., showed that the viability of sperm was markedly reduced in sperm separated from the ejaculate or epididymis in bovines. At the same time, there was a decrease in mitochondrial membrane potential (MMP) and a fragmentation of paternal DNA when exposed to AFB_1_ at doses of 1, 10, and 100 μM, for 4 h [56]. It is widely recognized that MMP, which reflects the function and energy state of mitochondria, has a connection with sperm motility and viability in mammals [82]. The MMP forms a proton gradient that is crucial for ATP synthesis. Consequently, any reduction in MMP can lead to a decrease in ATP production in germ cells [83]. Moreover, the activities of mitochondrial respiratory chain complexes (ETCs) I, III, and IV, control both mitochondrial membrane potential and ATP production. Previous studies have proved that the activities of ETC complexes I, II, III, and IV, as well as ATP production, were significantly decreased in the testis tissues of male mice treated with 0.375, 0.75, or 1.5 mg AFB_1_/kg of body weight per day, for 30 days [59]. These findings indicates that mitochondrial dysfunction caused by AFB_1_ exposure may be partly due to the inhibition of mitochondrial ETC activities and MMP in testicular tissue, emphasizing the importance of these mechanisms in regard to male reproductive toxicity.

Mitochondrial biosynthesis and dynamics also play an important role in regulating the functionality of mitochondria [84]. Mitochondrial biogenesis ensures the renewal and adaptation of the mitochondrial population when there is damage or an increased need for energy; if biogenesis is impaired, mitochondrial dysfunction can be worsened [85]. Huang et al. discovered that when AFB_1_ was orally administered at doses of 0.375, 0.75, or 1.5 mg AFB_1_/kg of body weight per day for 30 days, the expression of nuclear respiratory factor 1 (Nrf1), peroxisome proliferator-activated receptor gamma coactivator-1α (PGC-1α), and transcription factor mitochondrial transcription factor A (TFAM) proteins, were significantly downregulated [59,61]. PGC-1α is regarded as the main regulator of mitochondrial biogenesis and function; both in vivo and in vitro, its overexpression can enhance mitochondrial biogenesis and boost mitochondrial respiratory chain activities [86]. PGC-1α could transcriptionally activate the expression of Nrf2 and Nrf1 [86]. Furthermore, Nrf1 regulates the transcription of TFAM, then binds to mitochondrial DNA (mtDNA) and starts the expression of mtDNA gene products encoding subunits of ETC enzyme complexes and regulates the function of the mitochondrial respiratory chain [87]. Available evidence indicates that the downregulation of the PGC-1α/Nrf1/TFAM pathway by AFB_1_ resulted in a reduction in mitochondrial biosynthesis. Nrf1 cooperates with DNA methylation and directly regulates germ cell-specific genes, such as ankyrin repeat, SAM, and basic leucine zipper domain-containing 1 (ASZ1), and plays a critical role in the process of spermatogenesis [88]. This indicates that the inhibition of the PGC-1α/Nrf1 pathway may also contribute to the dysfunction of spermatogenesis. In addition, previous studies have also found that AFB_1_ exposure could significantly downregulate the expression of the p-AMPK protein in primary Leydig cells and the testis tissues in adult rats exposed to AFB_1_ [46]. AMPK is a key regulator of PGC-1α signaling and the activation of AMPK positively regulates the expression of PGC-1α, which promotes mitochondrial biogenesis [89]. It has been demonstrated that the inhibition of AMPK/PGC-1α contributes to drug or high-fat diet (HFD)-induced testicular injury [89]. This pathway was also documented to participate in AFB_1_-induced liver toxicity [51]. Therefore, mitochondrial biosynthesis in testicular tissues as a result of AFB_1_ exposure may also involve the inhibition of the AMPK/PGC-1α pathway. But precious mechanisms still need to be addressed. Moreover, in the testicular tissues of animals treated with AFB_1_, the expression of several biomarkers of mitochondrial dynamics, such as fission 1 (Fis1), optic atrophy 1 (Opa1), dynamin-related protein 1 (Drp1), and mitofusins 1 (Mfn1), were significantly decreased [90]. Mitochondrial dynamics consist of the opposing processes of fusion and fission, and under normal physiological conditions, they are generally balanced. When this balance is disrupted, the excessive production of ROS will occur, which leads to lipid peroxidation, a decrease in MMP, and reduced respiratory function and ATP production, finally culminating in mitochondrial dysfunction [90]. Collectively, these results indicate that exposure to AFB_1_ may damage mitochondrial dynamics, thus promoting testicular mitochondrial dysfunction.

A key biomarker of mitochondrial dysfunction is the release of cytochrome C (CytC) from mitochondria into the cytoplasm through the mitochondrial permeability transition pore (MPTP), coupled with an elevated Bcl2-associated X (Bax)/B-cell lymphoma-2 (Bcl-2) ratio [91,92]. These two occurrences might initiate apoptosis by activating caspases-9 and -3 [6]. Correspondingly, acute exposure of sheep to AFB_1_ at a dose of 1 mg/kg of body weight, via oral intake, significantly reduced the expression of the anti-apoptotic protein Bcl-2 and increased the Bax/Bcl-2 ratio, eventually resulting in enhanced apoptotic cell death in testicular tissue [39]. Chen et al. discovered that in rat testes, exposure to AFB_1_ at doses of 1.5, 15, or 150 μg/mL could upregulate the expression of Bax in a dose-dependent manner, while downregulating the expression of the Bcl-2 protein [46]. Likewise, a number of studies have recorded that AFB_1_ exposure could upregulate the expression of CytC, caspase-9, caspase-3, cleaved caspase-3, and p53 mRNAs and proteins, in the testes and epididymis of mice and rats in a dose- and time-dependent manner [27,33,38,93]. Suppressing oxidative stress and mitochondrial dysfunction by providing antioxidant supplements effectively improves AFB_1_-induced mitochondrial apoptotic pathways in the testicular tissues of animals, highlighting the crucial functions of oxidative stress and mitochondrial dysfunction in AFB_1_-induced toxicity [61].

**Figure 3:**
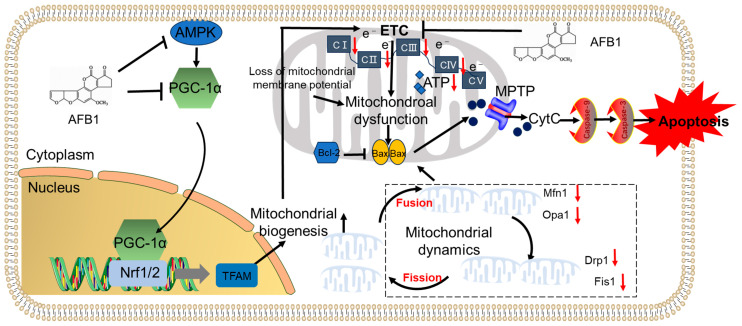
A schematic summary of the mechanisms of AFB_1_-induced mitochondrial dysfunction in testicular tissues. Notes: AFB_1_, aflatoxin B_1_; AMPK, adenosine 5′-monophosphate-activated protein kinase; Bcl-2, B-cell lymphoma-2; Bax, Bcl2-associated X; CytC, cytochrome c; ETC, mitochondrial respiratory chain complex; Fis1, fission 1; Mfn1, mitofusins 1; PGC-1α, peroxisome proliferator-activated receptor gamma coactivator-1α; TFAM, transcription factor mitochondrial transcription factor A; MPTP, mitochondrial permeability transition pore.

In summary, exposure to AFB_1_ can result in mitochondrial dysfunction within testicular tissues. Subsequently, this is capable of triggering apoptosis via the activation of the mitochondrial apoptotic pathway. The potential mechanisms underlying AFB_1_-induced mitochondrial dysfunction may involve suppressing mitochondrial biosynthesis, reducing respiratory chain activity, and disturbing mitochondrial dynamics (Figure 3). Furthermore, AFB_1_’s suppression of mitochondrial biosynthesis could be associated with the inhibition of the PGC-1α/Nrf1/2/TFAM pathway. Additionally, AFB_1_-induced inhibition of AMPK may influence mitochondrial biosynthesis through the PGC-1α pathway. Importantly, these findings provided new therapeutic targets for ameliorating AFB_1_-induced male infertility.

### 4.3. AFB_1_-Related Induction of Inflammatory Responses

Inflammation represents a critical contributing factor to male infertility [94]. Previous research has shown that inhibiting inflammatory responses is a crucial pathological characteristic in AFB_1_-induced hepato-, nephro-, and neurotoxicity [6,7,78]. Several investigations have reported that AFB_1_ exposure elicits inflammatory responses in the testicular tissues of animals [25,26,40,60,62,63]. NF-κB is a vital transcription factor that governs the expression of diverse pro-inflammatory genes, such as tumor necrosis factor alpha (TNF-α), interleukin-1 beta (IL-1β), IL-6, and cyclooxygenase-2 (COX2) [95,96,97]. In general, NF-κB can be activated by various conditions and factors, like the MAPK pathway, DNA damage, and excessive ROS [98]. Ijaz et al. discovered that AFB_1_ exposure at a dose of 50 μg/kg of body weight, per day, for 56 days, notably triggered the transcriptional activation of NF-κB, leading to the upregulation of IL-1β, IL-6, COX2, and TNF-α mRNAs and proteins in the testicular tissues of rats [27]. MAPKs consist of three classic pathways: the c-Jun amino-terminal kinase (JNK) pathway, the extracellular signal-regulated kinases 1 and 2 (ERK1/2) pathway, and the p38 pathway. All of these can induce inflammatory responses, either directly or indirectly, by activating nuclear NF-κB [98]. For example, the p38 pathway can prompt the upregulation of the nuclear NF-κB protein via the activation of mitogen- and stress-activated kinase 1/2 (MSK1/2), and the targeted inhibition of p38 signaling can effectively diminish inflammatory responses [99]. Recently, Zhang et al. reported that AFB_1_ exposure at a dietary dose of 0.5 mg/kg of feed (approximately equivalent to 0.3 μg/kg of body weight per day), for 21 days, significantly increased the protein levels of TNF-α, IL-1β, and IL-6, while significantly decreasing IL-10 in the testicular tissues of rabbits. Furthermore, they found that AFB_1_ exposure strikingly upregulated the expression of p38, MSK1/2, and NF-κB mRNAs and proteins in the testicular tissues of rabbits [40]. These findings imply that the activation of the p38/MSK1/2/NF-κB pathway plays a substantial role in the inflammatory response induced by AFB_1_ exposure in testicular tissues. In another study, it was reported that AFB_1_ exposure considerably upregulated the expression of IL-6, TNF-α, and NLRP3 proteins in the testes of mice [26]. NLRP3, which can activate inflammatory responses, serves as an important connection in the NOD-like receptor signaling pathway. It was selected to measure the association between inflammation and NOD-like receptor signaling, NLRP3, which is capable of activating inflammatory responses, and serves as an important link in the NOD-like receptor signaling pathway [7]. Previous studies have proven that the activation of the NLRP3 pathway contributes to the inflammatory responses induced by AFB_1_ exposure in liver and brain tissues [7]. Thus, it is suggested that the NLRP3 pathway also plays a part in the inflammatory response induced by AFB_1_ exposure in testicular tissues. Furthermore, crosstalk exists between the Nrf2 and NF-κB pathways; inhibition of the Nrf2/HO-1 pathway may lead to the activation of NF-κB [91]. Considering that AFB_1_ exposure significantly downregulates the Nrf2/HO-1 pathway in testicular tissues, this may partly contribute to the activation of the NF-κB pathway. The findings by Ijaz et al. illustrate that the activation of the Nrf2/HO-1 pathway through genkwanin supplementation markedly reduced the transcriptional activation of NF-κB and the expression of its downstream genes, such as IL-1β, IL-6, COX2, and TNF-α [27].

**Figure 4:**
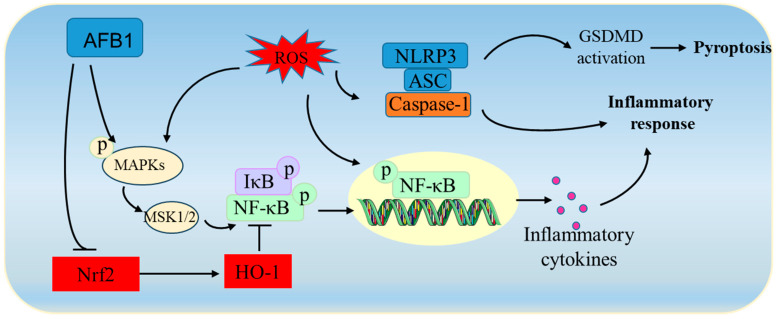
A schematic summary of the AFB_1_-induced inflammatory responses in testicular tissues. Notes: AFB_1_, aflatoxin B_1_; ASC, apoptosis-associated speck-like protein containing a CARD; GSDMD, gasdermin D; MAPKs, mitogen-activated protein kinases; MSK1/2, mitogen- and stress-activated kinase 1/2; NLRP3, NLR family pyrin domain-containing 3; Nrf2, nuclear factor erythroid 2-related factor 2; ROS, reactive oxygen species; HO-1, heme oxygenase-1.

In summary, current research data indicate that AFB_1_ exposure could induce inflammatory responses in testicular tissue via the activation of MAPKs, NF-κB, and NLRP3 pathways (Figure 4). The upregulation of ROS and the inhibition of Nrf2 due to AFB_1_ exposure may also contribute to the activation of inflammatory responses via the regulation of NF-κB. But the previously mentioned molecular mechanisms still need to be investigated further. Additionally, NLRP3-mediated pyroptosis, via the activation of gasdermin D (GSDMD), has been demonstrated to be involved in AFB_1_ exposure-induced hepato- and neurotoxicity [7,78]. Recently, a transcriptomics study revealed that multiple innate immune regulatory pathways are involved in regulating AFB_1_ exposure-induced reproductive toxicity, such as the NOD-like receptor signaling pathway, cytosolic DNA-sensing pathway, and RIG-I-like receptor signaling pathway [63]. These pathways have been implicated in AFB_1_ exposure-induced hepato- and nephrotoxicity [6,7]. Further exploration is needed to determine how these inflammatory signaling pathways, regulating reproductive toxicity, are impacted by AFB_1_ exposure.

### 4.4. AFB_1_-Related Induction of Autophagy

Autophagy plays a crucial role in maintaining cellular homeostasis. Autophagosome formation involves the development of double-membrane structures that engulf cytoplasmic material, which subsequently fuse with lysosomes for degradation [100]. A great number of studies have emphasized that abnormal autophagy can result in acrosome biogenesis flaws and spermatid differentiation issues during spermatogenesis, decreased serum testosterone levels, mitochondrial dysfunction, apoptotic cell death in Sertoli cells, and damage to the blood–testis barrier [101]. LC3, Belcin1, and p62 are three classic biomarkers of autophagy [100]. Of note, LC3 functions as the most dependable biomarker of autophagy and is essential for autophagosome formation. Beclin1 is a recruitment prerequisite for autophagosome formation. P62, an endogenous autophagy substrate, binds LC3 and ubiquitin to promote the autophagy of polyubiquitinated proteins and is inversely related to autophagy flux [101]. Huang et al. found that treating primary goat Sertoli cells with AFB_1_, at a dose of 50 μM for 24 h, significantly promoted the formation of autophagosomes. Moreover, they noticed that when primary goat Sertoli cells were exposed to AFB_1_ at doses of 10, 20, and 50 μM for 24 h, the expression of the autophagy proteins LC3 and Beclin1 were remarkably upregulated and the expression of p62 was downregulated [49]. Similarly, Chen et al. discovered that when rats were orally administered with AFB_1_, at a dose of ≥15 μg/kg of body weight, the expression of Beclin1, LC3, p62, and p-mTOR proteins were significantly upregulated and the expression of p-AMPK was significantly downregulated in the testis tissues [46]. Generally, increased levels of LC3 or Beclin1 confirm the activation of autophagy, which implies that AFB_1_ exposure can activate autophagy. Additionally, researchers found that treating primary adult rat Leydig cells with chloroquine (an autophagy inhibitor) significantly worsened the AFB_1_ exposure-induced expression of LC3II [46], suggesting that AFB_1_ blocks autophagy flux. They also found that rapamycin, an mTOR inhibitor, effectively alleviated AFB_1_ exposure-induced cytotoxicity in primary adult rat Leydig cells and primary goat Sertoli cells [46,49], indicating that mTOR pathway-mediated autophagy activation plays a vital role in AFB_1_ exposure-induced male reproductive toxicity. Significantly, mTOR can be regulated by multiple signaling molecules or pathways, such as oxidative stress, AKT, and AMPK. Guo et al., reported that AFB_1_ exposure, at 0.5 μM for 24 h, could significantly regulate the expression of transient receptor potential mucolipin 1 (TRPML1), an ion channel protein located on the lysosomal membrane [63]. It is reported that TRPML1 links lysosomal calcium to autophagosome biogenesis [34]. The inhibition of oxidative stress could significantly downregulate the expression of TRPML1 induced by AFB_1_ exposure, then reduce the activation of autophagy [34], indicating that the ROS/TRPML1 pathway partly participated in AFB_1_ exposure-induced autophagy. It has been reported that AFB_1_ exposure affects the expression of p-AMPK and p-AKT proteins, potentially regulating mTOR-mediated autophagy [46,49,63]. The capacity of various small molecules to target AKT and AMPK proteins in order to regulate autophagy could provide a new treatment strategy for AFB_1_ exposure-mediated male reproductive toxicity.

### 4.5. AFB_1_-Related Induction of Cell Cycle Arrest

Several recent studies have shown that male reproductive toxicity caused by AFB_1_ is related to cell cycle arrest [26,57,102]. Zhang et al. indicated that when mice were orally exposed to AFB_1_, at doses of 100, 200, and 400 μg/mL for 28 days, the expression of meiosis-related proteins like the Deleted in Azoospermia-like (DAZL) protein, synaptonemal complex protein 1 (SYCP1), and SYCP3, was significantly reduced. This shows that spermatocyte meiosis in the testes of mice was disrupted [102]. In addition, AFB_1_ exposure decreased the expression of cyclin A1 (CCNA1), cyclin B1 (CCNB1), and cyclin-dependent kinase D1 (CDK1) proteins, in mouse testis tissue and porcine Sertoli cells, leading to cell cycle arrest during the G2 phase in Sertoli cells [102]. Similarly, Zamir-Nasta et al. discovered that when mice were intraperitoneally (i.p.) administered with AFB_1_, at a daily dose of 20 µg/kg of body weight for 7, 14, or 21 days, cyclin D1, cyclin-dependent kinase D4 (CDK4), and estrogen receptor α (Erα) expression at both mRNA and protein levels were reduced, while p21 and p53 mRNAs and proteins, in a time-dependent manner, in the testicular tissue, were upregulated [57]. P53 and p21 play crucial roles in various biological processes, such as apoptosis, ferroptosis, cell cycle regulation, development, cell differentiation, cuproptosis, and senescence [103,104]. Targeted inhibition of p53 or gene deletion has effectively alleviated AFB_1_-induced cytotoxicity in HepG2 cells and mouse liver toxicity [105]. Moreover, Huang et al. found that AFB_1_ exposure could lead to S phase arrest by increasing p21 expression in HEK293T cells [58]. These results imply that the p53 or p21 signaling pathway may be involved in male reproductive toxicity caused by AFB_1_ exposure and further in-depth research is needed.

The Ras/PI3K/Akt signaling pathways are important mechanisms for controlling the cell cycle, proliferation, and survival, in response to extracellular signals [106]. It has also been reported that Ras and its downstream signals, including PI3K, are related to sperm motility. Furthermore, it was reported that AFB_1_ exposure could effectively block Ras/PI3K/AKT signaling pathways [102]. Wang et al. confirmed that the downregulation of CDK1 can result in decreased AKT phosphorylation and cause cell cycle arrest [106]. These findings suggest that AFB_1_ exposure may interfere with testicular development through cell cycle-related Ras/PI3K/Akt signaling pathways.

### 4.6. Role of Gut Microbiota in AFB_1_-Related Toxicity

Recent studies suggest that AFB_1_ can drive changes in gut microbiota, influence the peripheral inflammatory environment, and contribute to metabolic disorders, all of which may affect testicular function [26,102]. For instance, Zhang et al. found that oral exposure to AFB_1_ induces alterations in the gut microbiome composition and disrupts the balance of serum metabolites [102]. Comparative analyses have indicated that these changes are associated with the regulation of the Ras signaling pathway, which appears to play a critical role in AFB_1_-induced testicular development [102]. Significantly, the supplementation of an isolated probiotic, Bacillus licheniformis, isolated from pig manure, effectively mitigated AFB_1_-induced disruptions in gut flora. This intervention subsequently alleviated testicular developmental damage via the inhibition of inflammatory responses and oxidative stress, while upregulating the AKT signaling pathway [26]. In another study, it was found that probiotic supplementation could effectively improve AFB_1_ exposure-induced oxidative stress, mitochondrial dysfunction, and apoptosis, in mouse Sertoli TM4 cells, via the upregulation of the Nrf2 pathway and AKT/mTOR-mediated autophagy pathway and the inhibition of ROS production [63]. These findings illuminate that the gut microbiota and gut–testis axis are involved in AFB_1_-induced reproductive toxicity. The potential mechanisms may involve the regulation of oxidative stress, inflammatory responses, AKT/mTOR-mediated autophagy, and apoptosis. However, the precise molecular mechanisms remain incompletely understood.

## 5. Chemo-Protective Agents Against AFB_1_-Induced Male Reproductive Toxicity

It is critical to comprehend the molecular mechanisms underlying the reproductive toxicity of aflatoxin, as it will establish the necessary theoretical foundation and will help to identify intervention targets for chemical prevention and detoxification. This understanding offers potential for the development of more effective treatment strategies. Although the precise molecular mechanisms of AFT-induced reproductive toxicity remain unclear, numerous studies have demonstrated the involvement of oxidative stress, autophagy, inflammatory responses, and various signaling pathways, in the regulation of male reproductive toxicity caused by aflatoxin. It has been found that a few small molecular compounds (e.g., caffeic acid, selenium, rapamycin, silymarin, and genkwanin), Chinese herbal extracts (e.g., Sorghum bicolor L. Moench), hydrophobic fractions (enriched in apigenin), probiotic supplementations (e.g., ZG7, Lactobacillus casei, Bacillus subtilis, and Saccharomyces cerevisiae), and camel milk, could effectively improve AFB_1_-induced male reproductive toxicity by targeting the aforementioned signaling pathways [25,26,27,33,46,54,55,63].

Table 2 provides a summary of the protective effects of a diverse array of compounds against AFB_1_-induced male reproductive toxicity. These findings offer crucial treatment strategies regarding male reproductive toxicity and infertility induced by AFTs. Nevertheless, these findings have only been verified via preliminary animal models and more sub-clinical and clinical trials are required to confirm the findings. Moreover, previous studies have also reported that some adsorbent materials (such as montmorillonite clay), chemical methods (such as ozone), and natural plant extracts (such as artichoke leaf extract, mahua (Madhuca indica Gmel.) seeds, and Chinese gallnut tannic acid), could potentially offer protection against AFB_1_-induced toxic effects in vivo and in vitro [107,108,109,110]. However, answering the question as to whether these methods could provide effective protection against AFB_1_-induced male reproductive toxicity still requires more experimental and clinical studies. Given the significance of male reproductive health and the increasing prevalence of infertility issues in modern society, further exploration of these potential treatment strategies is of utmost importance.

## 6. Conclusions and Future Directions

Reproductive toxicity represents a crucial adverse impact resulting from exposure to AFTs, especially AFB_1_. In animal experiments, it has been found that continuous exposure to AFB_1_ allows the toxin to penetrate the blood–testis barrier and poison testicular tissues. Such seeping leads to irreversible histological and pathological damage, which manifests as decreased testicular function. The reproductive toxicity caused by AFB_1_ is related to multiple mechanisms, like oxidative stress, cell cycle arrest, apoptosis, inflammatory reactions, and autophagy. Numerous signaling pathways are involved, namely Nrf2, mTOR, PI3K/AKT, PGC-1α, AMPK, p53, MAPK, NF-κB, STAR, NLRP3, and mitochondrial apoptotic pathways. These results highlight important target protective agents against AFB_1_-induced reproductive toxicity. To date, various small molecular compounds (i.e., caffeic acid, selenium, rapamycin, silymarin, and genkwanin), Chinese herbal extracts (for example, the hydrophobic fraction of Sorghum bicolor *L. Moench* rich in apigenin), probiotic supplements (i.e., ZG7, Lactobacillus casei, Bacillus subtilis, and Saccharomyces cerevisiae), and camel milk, have been recognized as effective substances for reducing AFB_1_-induced male reproductive toxicity. The current findings suggest several directions for future research: (1) Human clinical trials and animal experiments are required to enhance the reproductive protection effects of these natural products, compounds, and probiotics. (2) It is necessary to investigate the relationship between the environmental exposure to AFTs and male infertility. (3) The exact molecular mechanisms according to which the aforementioned dietary supplements protect against AFB_1_-induced reproductive toxicity need further study. (4) We need to further distinguish the main mechanisms of action of probiotics or camel milk in fighting against the toxic effects of AFB_1_ exposure, especially whether their effectiveness comes from AFB_1_ degradation alone or from targeting key host proteins to make the host resistant to AFB_1_ toxicity. In conclusion, we hope this review offers useful ideas and inspires more extensive discussions for developing effective protective agents against male reproductive toxicity caused by AFT exposure.

## Figures and Tables

**Table 1:** A summary of AFB_1_ exposure-caused detrimental effects on the reproductive organs and performance of male animals.

Animals	Doses	Administration Time	Administration Route	Toxic Effects	References
White leghorn male birds	AFB_1_ mixed in feed containing 100, 200, or 400 ppb	10 weeks	Diet	In the birds fed with ABF_1_, a reduction in testicular size and volume was detected. Meanwhile, through histological examinations, it was found that there was moderate-to-severe necrosis in the testicular parenchyma and spermatogenesis was partially or completely arrested.	[47]
Rats	30 μg/kg of body weight per day	3 days per week for 6 weeks	Orally	AFB_1_ exposure resulted in a marked decrease in the relative weight of the testes and sperm count. Furthermore, exposure to AFB_1_ led to significant histopathological alterations in testicular tissue. This was manifested as a reduction in the thickness of the epithelium lining, moderate diffuse degeneration, and vacuolation of Sertoli cells. Additionally, marked spermatogenic damage was also detected.	[48]
Male Kunming mice	0.375, 0.75, or 1.5 mg/kg of body weight per day	30 days	Orally	Exposure to AFB_1_ resulted in a reduction in the relative weight of the testis, the sperm count within the epididymis, and the motility of sperm. Moreover, AFB_1_ exposure can also lead to a decrease in the expression of junction proteins related to the blood–testis barrier, trigger mitochondrial damage, and increase apoptosis in Sertoli cells and the testes. These effects are associated with oxidative stress-mediated inhibition of the Akt/mTOR pathway and the activation of p38 MAPK signaling. In testis tissues treated with AFB_1_, atrophic seminiferous tubules, vacuole-like changes in the spermatogenic epithelium, along with a decrease in Leydig cells and sperm numbers, can be detected.	[38,49]
Mice	Dose of 1500, 375, or 93.75 μg/kg of body weight per day	50 days	Orally	In all the treatment groups, the relative weight of the testis and sperm number were significantly decreased. In addition, the sperm deformity rate was increased in all AFB_1_-treated groups. AFB_1_ treatment at doses of 375 and 1500 μg/kg of body weight per day for 50 days could result in marked DNA damage in the testis tissues.	[50]
Sheep	1.0 mg/kg of body weight	Signal dose	Orally	Following AFB_1_ treatment, degeneration, intracellular vacuole formation was detected, along with a decrease in the diameter of the vas deferens and a notable thinning of the spermatogenic epithelium. Additionally, testosterone concentrations in the testicles were significantly increased.	[39]
Mice	50 µg/kg of body weight per day	28 days	Intraperitoneal injection	AFB_1_ treatment significantly decreased the relative weight of the testes and epididymis, decreased the function of sperm (such as motility, viability, and count), and increased the rate of sperm abnormalities. Obvious apoptosis and inflammatory responses were observed in AFB_1_-treated testicular tissue.	[25,33]
Rats	0.5 mg per kg of body weight	7 days	Orally	AFB_1_ exposure can result in an overabundance of vacuolar cells and the inhibition of spermatogenesis in the testis.	[52]
Rats	Dose of 7.5 μg/200 g of body weight/16 days	16 days	Orally	Upon being treated with AFB_1_, the normal structure of the spermatogenic cells worsened and the seminiferous tubules became necrotic and underwent degeneration. Additionally, AFB_1_ treatment reduced sperm motility and viability, and triggered cell apoptosis and lipid peroxidation in the testicular tissue.	[53]
Mice	0.75 mg/kg of body weight per day	45 days	Orally	AFB_1_ treatment significantly decreased the testes index and sperm function parameters (including concentration, malformation, and motility). Marked oxidative stress damage in testicular tissues was also detected in AFB_1_-treated mice.	[54]
Rabbits	0.5 mg/kg of feed and 0.3 μg/kg of body weight AFB_1_ was consumed by the rabbits daily	21 days	Diet	After AFB_1_ treatment, the testicular coefficient was significantly decreased. In addition, obvious changes in the morphological structure (such as a decreases in the diameter, area, and thickness of spermatogenic tubules, and the thickness of the spermatogenic tubule epithelium) were detected. AFB_1_ treatment also caused spermatogenesis disorders, which was evident due to decreases in plasma sex hormones and sperm.	[40]
ICR mice	200 μg/kg of body weight per day	28 days	Orally	After AFB_1_ exposure, obvious histopathological changes and abnormal morphology of sperm were found in the testicular tissue. The protein expression of sex hormones and hormone synthesis were significantly downregulated. AFB_1_ exposure also triggered oxidative stress and inflammatory damage in testicular tissue.	[26]
Rats	1.5, 15, or 150 μg/kg of body weight per day	21 days	Orally	Compared with the control group, AFB_1_ did not have an impact on the weight of the rat’s body, testis, or epididymis. However, it led to a significant reduction in the levels of serum hormones (such as testosterone, LH, and FSH levels) and the number of Leydig cells.	[46]

**Table 2:** A summary of the protection effects of diverse compounds regarding male reproductive toxicity triggered by AFB_1_ exposure in vivo or in vitro.

Natural Products/Bioactive Substances	Models	Doses	Administration Route	Administration Time	The Protective Effects	Reference
Caffeic acid	Rats	40 mg/kg of body weight/day	Orally	28 days	Caffeic acid supplementation has the potential to ameliorate, in a dose-dependent manner, oxidative stress, inflammatory responses, and the activation of apoptotic signals in testicular and epididymal tissues of rats induced by AFB_1_ exposure. Moreover, it can enhance the functional properties of spermatozoa and reproductive hormone levels, as well as avert the histological changes brought about by AFB_1_ exposure.	[25]
Selenium	Mice	0.2 or 0.4 mg/kg of body weight per day	Orally	45 days	In AFB_1_-exposed mice, selenium supplementation led to an increase in the testes index, sperm functional parameters, including concentration, malformation, and motility, as well as the level of serum testosterone. Additionally, the supplementation of selenium lessened the oxidative stress induced by AFB_1_ and mitigated the reduction in the protein expression of testicular testosterone synthesis enzymes like STAR, P450 side-chain cleavage (P450scc), and 17β–HSD, in these mice.	[54]
Genkwanin	Rats	0 mg/kg of body weight/days	Orally	8 weeks	When compared to rats treated solely with AFB_1_, genkwanin supplementation increased the sperm count, decreased the sperm deformity rate, and enhanced the levels of gonadotropins and plasma testosterone. Moreover, genkwanin supplementation alleviated AFB_1_-induced oxidative stress, inflammatory responses, and the activation of the mitochondrial apoptotic pathway, and reduced the decrease in the gene expression of testicular testosterone synthesis enzymes, such as HSB17b, HSB3b, STAR, CYP11A1, and CYP17A, in the testes of AFB_1_-exposed rats.	[27]
Sorghum bicolor (L. Moench) hydrophobic fraction (enriched in apigenin)	Rats	5 or 10 mg/kg of body weight/days	Orally	28 days	The supplementation of Sorghum bicolor (L.) Moench (SBE-HP) extract can remarkably restrain the oxidative, inflammatory, apoptotic, and histological disorders caused by AFB_1_. It achieves this by enhancing sperm function parameters, testicular enzymes, and reproductive hormones.	[33]
Camel milk and silymarin	Rats	1 mL camel milk/kg of body weight/day	Orally	21 or 28 days	Camel milk and silymarin supplementation could effectively ameliorate AFB_1_ exposure-induced decreases in serum testosterone, testes pathology changes. They also significantly downregulated the expression of TNF-α and upregulated the expression of testosterone synthesis genes (including LHR and STAR), in the testes of rats.	[55]
ZG7 (a stain of Bacillus licheniformis)	Mice	2 μL/g of body weight per day	Orally	28 days	ZG7 supplementation has remarkable AFB_1_ degradation efficiency. In addition, ZG7 supplementation ameliorated AFB_1_ exposure-caused testicular dysplasia, which was evident through the improvement of the sperm concentration and the increase in the expression of testis development-related proteins (including CYP17A1, CYP11A1, γH2AX, SYCP3, and GJA1) and the decrease in oxidative stress and inflammatory biomarkers.	[26]
Rapamycin	Primary adult Leydig cell	100 μM	Cell culture	24 h	Rapamycin treatment induced autophagy, then markedly inhibited AFB_1_-induced oxidative stress, the activation of the mitochondrial apoptotic pathway, and cell apoptosis.	[46]
Compound probiotics (including L. casein, B. subtilis, and S. cerevisiae, a ratio of 1:1:1)	Mouse TM4 cells	At a concentration of 1.0 × 109 CFU/mL	Cell culture	24 h	The supernatant obtained from the supplementation of compound probiotics can remarkably suppress the generation of ROS (reactive oxygen species) and cell apoptosis caused by AFB_1_ exposure. Additionally, it significantly decreases the quantity of autophagic vacuoles, activates the Nrf2 pathway, and restrains the activation of the mitochondrial apoptotic pathway within TM4 cells.	[63]
